# Impact of total cerebral small vessel disease score on ophthalmic artery morphologies and hemodynamics

**DOI:** 10.1186/s12967-023-03908-y

**Published:** 2023-02-01

**Authors:** Jia-lin Wang, Xue-ru Cheng, Zhao-yang Meng, Yan-ling Wang

**Affiliations:** Department of Ophthalmology, Beijing Friendship Hospital, Capital Medical University, 95 Yong’an Road, Xicheng District, Beijing, 100050 China

**Keywords:** Cerebral small vessel disease, Ophthalmic artery, Computational fluid dynamics

## Abstract

**Background:**

Cerebral small vessel disease (CSVD) is a systemic disease, affecting not only the brain, but also eyes and other organs. The total CSVD score is a tool for comprehensive evaluation of brain lesions in patients with CSVD. The ophthalmic artery (OA) is a direct response to ocular blood flow. However, little is known about the correlation between CSVD and characteristics of OA. We investigated the OA morphologies and hemodynamics in patients with CSVD and the correlation between these changes and the total CSVD score.

**Methods:**

This cross-sectional observational study included 34 eyes from 22 patients with CSVD and 10 eyes from 5 healthy controls. The total CSVD score was rated according to the CSVD signs on magnetic resonance imaging. OA morphological characteristics were measured on the basis of 3D OA model reconstruction. OA hemodynamic information was calculated using computational fluid dynamics simulations.

**Results:**

The total CSVD score negatively correlated with the OA diameter, blood flow velocity, and mass flow ratio (all P < 0.05). After adjusting for potential confounding factors, the total CSVD score was still independently correlated with the OA blood velocity (β = − 0.202, P = 0.005). The total CSVD score was not correlated with OA angle (P > 0.05). The presence of cerebral microbleeds and enlarged perivascular spaces was correlated with the OA diameter (both P < 0.01), while the lacunar infarcts and white matter hyperintensities were correlated with the OA blood velocity (both P < 0.001).

**Conclusions:**

The decrease of the blood velocity in the OA was associated with the increase in the total CSVD score. The changes of the OA diameter and velocity were associated with the presence of various CSVD signs. The findings suggest that more studies are needed in the future to evaluate CSVD by observing the morphologies and hemodynamics of OA.

## Introduction

Cerebral small vessel diseases (CSVDs) are a group of clinical and pathological syndromes with pathological mechanisms affecting the small vessels of the brain [1]. The prevalence increases with age, and CSVDs affect 5% of people over 50 years and approximately 100% of those aged over 90 years [2]. CSVD causes brain parenchymal lesions and cognitive impairment and accounts for 25% of stroke cases and 45% of dementia cases [2]. Stroke outcomes become worse in those with more severe CSVD-related brain parenchymal lesions [3–5].

Owing to the difficulty of visualizing cerebral small vessels, some brain parenchyma lesions that are closely correlated with small vessel changes have been regarded CSVD markers. To simplify the evaluation of CSVD, visual scoring systems based on magnetic resonance imaging (MRI) have been proposed [6]. Features on MRI subsumed by the total CSVD score mainly include lacunar infarcts, white matter hyperintensities (WMHs), cerebral microbleeds (CMBs), and enlarged perivascular spaces.

Retinal arteries and small cerebral arteries share similar characteristics. Both of them are terminal arteries with few anastomoses and similar diameters [7]. Many studies have reported the correlation between the severity of CSVD and retinal microvascular abnormalities. Kwa et al. found that retinal arterial narrowing and sclerosis were correlated with MRI signs of CSVD [7]. Lee et al. reported that retinal vascular density is negatively related to the CSVD score [8]. These results strongly suggest that ocular blood flow characteristics may be related to CSVD signs. However, there are no relevant reports on the morphologies and hemodynamics of the ophthalmic artery (OA) in CSVD. Most current studies have focused on the retinal microvasculature, which is easily affected by hypertension and diabetes mellitus. The OA was thought to directly reflect ocular blood flow [9]. Therefore, it is critical to analyze the correlation between CSVD signs and OA hemodynamics.

Digital subtraction angiography (DSA) and color Doppler imaging (CDI) are widely used to visualize the OA. However, DSA is invasive and may cause different degrees of complications, and it is difficult to capture the origin of the OA by using CDI. Currently, computed tomographic angiography (CTA) has been increasingly applied in neurovascular imaging, which clearly displays vascular morphologies with an increasing number of slice images.

We investigated the OA morphological and hemodynamic information in patients with CSVD by reconstructing the OA blood flow model and by using computational fluid dynamics (CFD) simulation. This study aimed to investigate the OA morphological and hemodynamic characteristics in patients with CSVD from the perspective of the total CSVD score. We hypothesized that the decreased diameter, lower blood velocity, and decreased blood flow of the OA would be associated with a higher total CSVD score.

## Methods

### Patients and data collection

This cross-sectional observational study was approved by the Medical Research Ethics Committee of Beijing Friendship Hospital, which is affiliated with Capital Medical University (2020-P2-008-01), and was conducted in accordance with the principles of the Declaration of Helsinki. Informed consent was obtained from all patients. The medical records of patients admitted to Beijing Friendship Hospital from November 2019 to March 2022 who underwent brain MRI and head and neck CTA for various reasons were reviewed. Brain MRI images were carefully examined, and each participant underwent a thorough ophthalmic examination. Patients who met any of the following criteria were excluded: (1) any cerebrovascular accident influencing the observation and evaluation of CSVD signs, including acute massive cerebral infarction or cerebral hemorrhage; (2) brain tumors; (3) no initial OA imaging visualized clearly on CTA images; and (4) any significant eye pathologies [9].

### Brain MRI acquisition and analysis

Brain MRI was performed using a GE Discovery MR750 3.0 T MRI scanner (GE Healthcare, Waukesha, WI, USA) with sequences including T1-weighted imaging, T2-weighted imaging, fluid-attenuated inversion recovery (FLAIR), diffusion-weighted imaging, and susceptibility-weighted imaging (SWI). The total CSVD score was rated on an ordinal scale from zero to four, according to the presence of CSVD signs on MRI. The rating criteria were as follows [6, 10]: (1) lacunar infarcts were defined as round or ovoid lesions with cerebrospinal fluid signal intensity on T2 and FLAIR, generally having a hyperintense rim on FLAIR, between 3 and 20 mm in diameter, and located in the territory of perforating arterioles. If there were one or more lacunar infarcts, one point was awarded. (2) WMH was identified as white matter lesions with high signal intensity on FLAIR and was assessed on the basis of the Fazekas scale [11]. One point was awarded if the deep WMH tended to fuse (Fazekas score reached 2 points) or the irregular periventricular WMH extended to the deep white matter (Fazekas score reached 3 points). (3) CMBs were identified as round or linear homogeneous lesions with low signal intensity on SWI and with a diameter of less than 5 mm. If there were one or more microbleeds, one point was awarded. (4) An enlarged perivascular space was defined as a round, ovoid, or linear lesion with the same intensity as lacunar infarcts but less than 3 mm in diameter. If the number of enlarged perivascular spaces in the basal ganglia was 10, 1 point was awarded. Two experienced clinicians independently evaluated the presence of CSVD signs on MRI. The total score was determined when the two independent scores for each patient were the same. If the scores differed, the two doctors worked together for re-evaluation.

### CTA data acquisition

CTA was performed from the aortic arch to the skull base by using a 64-row multidetector computed tomography scanner (LightSpeed VCT; GE Healthcare, Chicago, IL, USA). The contrast medium injection method and detailed scanning parameters were the same as those in our previous studies [9]. CTA image data were saved in Digital Imaging and Communications in Medicine (DICOM) format.

### Three-dimensional (3D) OA reconstruction

On the basis of the DICOM data of CTA images, a 3D model of the internal carotid artery (ICA) and OA was reconstructed in Mimics 21.0 (Materialise, Ann Arbor, MI, USA). Geomagic Studio 14.0 (3D Systems, Rock Hill, SC, USA) was used to smoothen the model surface. An appropriate edge length was set in remeshing to form a uniform triangular surface, which made subsequent operations more accurate. The diameter of the OA and the angle between the OA and the ipsilateral ICA were measured using Mimics 21.0. The diameter was measured from where the OA originated from the ICA. The entire process and results were evaluated and analyzed by two experienced ophthalmologists who were blinded to the clinical information of the patients.

### CFD simulation

After 3D reconstruction, ANSYS Fluent 19.0 (ANSYS, Inc., Canonsburg, PA, USA) was used for simulation calculation, with a finite-volume method for steady flow. The specific simulation steps are described in our previous study [9]. The simulated blood was presumed to be steady laminar incompressible Newtonian fluid. The numerical simulation was based on the Navier–Stokes equation and mass conservation equation:

1 $$ \rho (\vec{u} \cdot \nabla )\vec{u} + \nabla p - \mu \Delta \vec{u} = 0, $$

2 $$ \nabla \cdot \vec{u} = 0, $$

where ρ is blood density, u is velocity vector, p is pressure, and μ is blood viscosity. The material attribute values of blood were then set (µ = 3.5 × 10^−3^ kg/ms, ρ = 1050 kg/m^3^). The inlet velocity was set to 0.34 m/s, and the pressure boundary condition of the outlet was set to 0 Pa. The calculation ended when the fluid model converged. Subsequently, the fluid velocity streamline chart and mass flow values were created and calculated.

### Statistical analyses

SPSS Statistics 26.0 (IBM Corp., Armonk, NY, USA) was used for statistical analyses. P ≤ 0.05 indicated statistical significance. The Shapiro–Wilk test was performed for data normality. Data of normal continuous variables are presented as means ± standard deviations, while those of skewed variables are expressed as medians (25th percentile, 75th percentile). Categorical data are expressed as numbers (percentages). The t-test was performed for the comparison of normal continuous variables between the CSVD group and the HC group, and the Mann–Whitney U test was performed for the comparison of skewed variables between the two groups. For categorical data, the Fisher’s exact test was performed. The one-way analysis of variance with Bonferroni correction was performed for the comparison of the OA diameter and the blood velocity among multiple groups, and the Kruskal–Wallis *H* tests was performed for the comparison of the OA angle and the mass flow ratio among multiple groups. The univariate linear regression analysis and the multivariate regression analysis were performed to explore the correlation between the total CSVD score and the OA characteristics. Values of the OA blood flow velocities were transformed into natural logarithms in linear regression analysis.

## Results

### Patient baseline clinical characteristics

Thirty-four eyes of 22 patients with CSVD (mean age 63.88 ± 4.21; 29% female) and 10 eyes of 5 healthy controls (HCs) (mean age 62.20 ± 7.22; 20% female) were included in this study. Table 1 shows the baseline data of all participants. The mean systolic blood pressure (SBP) of patients with CSVD was significantly higher than that of the HC group (149.65 ± 17.61 mm Hg vs. 136.20 ± 18.15 mm Hg, P = 0.041). The differences in diabetes mellitus incidence rates (62% vs. 20%, P = 0.031) and the median hemoglobin A1c (HbA1c) (5.80% vs. 6.55%, P = 0.047) levels between patients with CSVD and those in the HC group were significant. There were no significant differences in age, sex, and other clinical characteristics between the two groups.

**Table 1:** Clinical Characteristics of Participants

Characteristics	HC (n = 10)	CSVD (n = 34)	P-value
Age (y), mean ± SD	62.20 ± 7.22	63.88 ± 4.21	0.356
Sex (female), n (%)	2 (20)	10 (29)	0.702
History, n (%)			
Smoking	6 (60)	15 (44)	0.481
Ischemic heart disease	2 (20)	13 (38)	0.452
Diabetes mellitus	2 (20)	21 (62)	0.031^*^
Hpertension	8 (80)	26 (77)	1.000
Dyslipidemia	8 (80)	26 (77)	1.000
Laboratory parameters			
SBP (mmHg), mean ± SD	136.20 ± 18.15	149.65 ± 17.61	0.041^*^
DBP (mmHg), median (P25, P75)	81.00 (78.50, 88.25)	82.00 (75.00, 88.00)	0.757
TC (mmol/L), median (P25, P75)	3.69 (3.52, 5.93)	3.77 (3.31, 4.17)	0.262
HDL (mmol/L), median (P25, P75)	0.99 (0.83, 1.03)	1.03 (0.89, 1.22)	0.103
LDL (mmol/L), median (P25, P75)	2.09 (1.86, 3.75)	2.20 (1.64, 2.47)	0.217
HBA1c (%), median (P25, P75)	5.80 (5.58, 5.93)	6.55 (5.63, 7.88)	0.047^*^
Homocysteine (μmol/L), median (P25, P75)	12.50 (11.05, 13.25)	13.50 (9.35, 17.53)	0.453

*SBP* systolic blood pressure, *DBP* diastolic blood pressure, *TC* total cholesterol, *HDL* high-density protein, *LDL* low-density protein, *HBA1c* hemoglobin A1c

^*^P < 0.05 is significant

### Morphological comparison

**Fig. 1:**
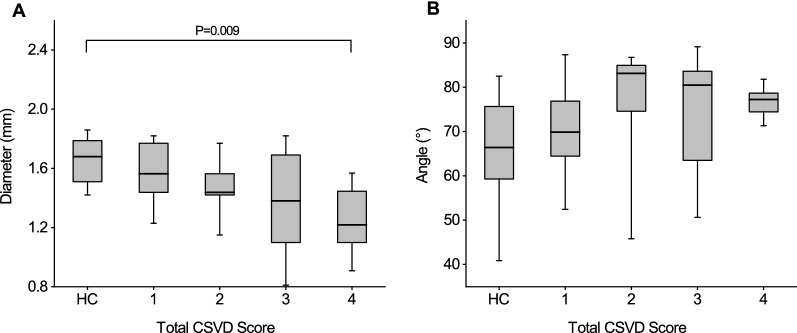
Comparison of the OA diameters (**A**) and angles (**B**) in the HC group and various CSVD score groups. The center lines in the boxes represent the median, the lower and upper ends of the boxes represent the 25th and 75th percentiles, respectively, and whiskers indicate the maximum and minimum values. The P-value was after Bonferroni correction

The mean OA diameters in the HC group and in the groups with the total CSVD scores of 1–4 points were 1.66 ± 0.16, 1.58 ± 0.21, 1.47 ± 0.18, 1.37 ± 0.35, and 1.25 ± 0.23 mm, respectively. The mean OA diameter in the group with a CSVD score of 4 points was significantly lower than that in the HC group (P = 0.009, after Bonferroni correction) (Fig. 1). The median OA angles in the HC group and in the groups with total CSVD scores of 1, 2, 3, and 4 points were: 66.36° (58.08°, 746.83°), 69.92° (63.18°, 78.36°), 83.13° (72.31°, 85.21°), 80.47° (62.44°, 84.42°), and 77.15° (73.97°, 78.78°), respectively. No difference in the OA angle was observed among the groups.

### Hemodynamic comparison

**Fig. 2:**
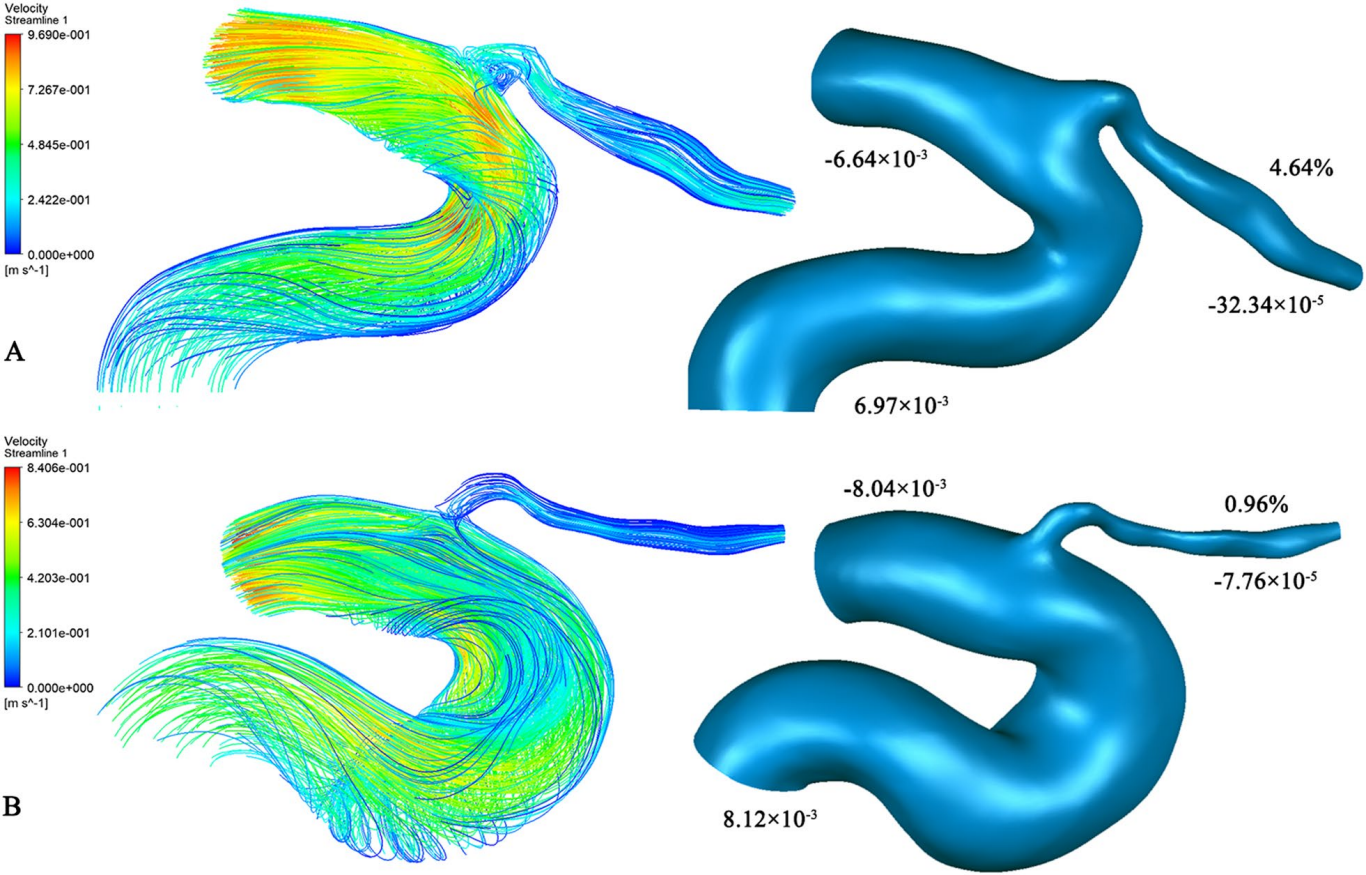
Blood flow streamline diagrams and mass flow diagrams in HCs (**A**) and CSVD (**B**). The streamline color indicates the blood velocity. The positive and negative values of mass flow (kg/s) represent the inlet and outlet respectively

**Fig. 3:**
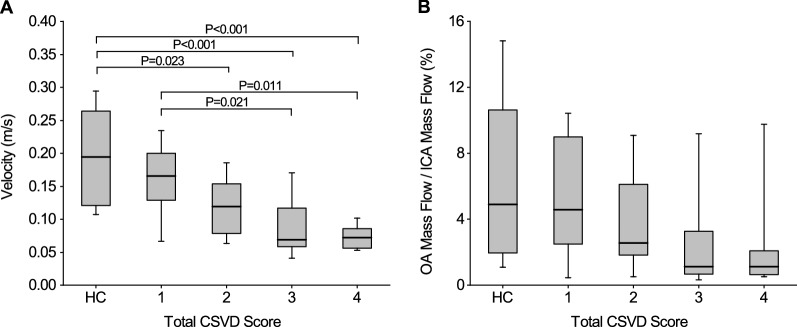
Comparison of the OA blood velocities (**A**) and the mass flow ratios (**B**) in the HC group and various CSVD score groups. The notes are the same as Fig. 1. P values were after Bonferroni correction

CFD simulation was performed to determine OA blood velocity and mass flow ratio of the OA to the ICA in each group (Fig. 2). Specifically, the mean OA blood velocities in the HC group and in the groups with total CSVD scores of 1, 2, 3, and 4 points were 0.197 ± 0.071, 0.161 ± 0.055, 0.119 ± 0.046, 0.083 ± 0.041, and 0.073 ± 0.018 m/s, respectively; moreover, the median mass flow ratios of these groups were 4.89% (1.93%, 10.66%), 4.57% (2.00%, 9.42%), 2.56% (1.61%, 7.42%), 1.13% (0.60%, 3.34%), and 1.11% (0.57%, 2.40%), respectively. Patients with CSVD had a lower blood flow velocity than those in the HC group (P < 0.05, after Bonferroni correction); however, no significant difference was found among groups in the mass flow ratio (Fig. 3).

### Correlation between the OA characteristics and the total CSVD score

In the linear regression analysis, the total CSVD score was negatively correlated with the OA diameter (β = − 0.107, P = 0.010), blood flow velocity (β = − 0.271, P < 0.001), and mass flow ratio (β = − 0.387, P = 0.018) in patients with CSVD, although it was not significantly correlated with the OA angle. A multivariate linear regression analysis was performed subsequently. After adjusting for sex, diastolic blood pressure, ischemic heart disease, and smoking, which were preliminarily considered blood flow velocity–related characteristics in the univariate correlation analysis, the association between the blood flow velocity and the total CSVD score remained significant (β = − 0.202, P = 0.005).

### Correlation of OA characteristics with specific CSVD signs and clinical parameters

Although there were differences in diabetes mellitus and the HbA1c levels between patients with CSVD and those in the HC group (Table 1), these factors were not associated with the OA diameter or blood velocity. As shown in Table 2, SBP, hemocysteine, WMHs, CMBs, and enlarged perivascular spaces were associated with the OA diameter in univariate linear regression. In the multivariate linear regression analysis, the presence of CMBs and enlarged perivascular spaces were still independently related to the OA diameter (P = 0.003 and P = 0.006, respectively). Ischemic heart disease, lacunar infarcts, and WMHs were independently related to the OA blood flow velocity in the multivariate linear regression analysis (P = 0.006, P < 0.001, and P < 0.001, respectively).

**Table 2:** The univariate linear regression analysis and multivariate stepwise regression analysis between clinical characteristics and OA diameter and blood flow velocity

		Univariate		Multivariate^a^	
	Variables	β (95% CI)	P Value^*^	β(95% CI)	P Value
Diameter	SBP	− 0.007 (− 0.011 to − 0.003)	0.001		
	Homocysteine	− 0.016 (− 0.030 to − 0.002)	0.025		
	WMHs	− 0.223 (− 0.375 to − 0.072)	0.005		
	CMB	− 0.263 (− 0.408 to − 0.118)	0.001	− 0.231 (− 0.380 to − 0.082)	0.003
	Enlarged perivascular spaces	− 0.245 (− 0.400 to − 0.090)	0.003	− 0.226 (− 0.382 to − 0.071)	0.006
Velocity^b^	Ischemic heart disease	− 0.529 (− 0.840 to − 0.219)	0.001	− 0.318 (− 0.539 to − 0.097)	0.006
	Lacunar infarct	− 0.663 (− 0.936 to − 0.391)	< 0.001	− 0.424 (− 0.648 to − 0.200)	< 0.001
	WMHs	− 0.709 (− 0.959 to − 0.459)	< 0.001	− 0.468 (− 0.692 to − 0.245)	< 0.001
	CMB	− 0.621 (− 0.892 to − 0.350)	< 0.001		

*SBP* systolic blood pressure, *WMHs* white matter hyperintensities, *CMB* cerebral microbleed

^*^P < 0.05 is significant in univariate linear regression analysis

^a^Significant variables in results of the univariate regression were included in the multivariate stepwise regression analysis

^b^Variables are transformed to natural logarithms

## Discussion

In this study, we examined the OA diameter and angle by reconstructing 3D models and calculating blood flow velocity and mass flow values by using CFD simulation. Furthermore, we assessed the participants’ total CSVD score and analyzed the correlation of the total CSVD score and the specific signs of CSVD with the OA morphological and hemodynamic characteristics.

Although there was no difference in the history of hypertension between the two groups, patients with CSVD had a significantly higher SBP than those in the HC group. Similarly, Chuang et al. reported that the CSVD score in patients with SBP > 140 mm Hg was more likely to increase than that in patients with SBP ≤ 140 mm Hg [12], thus supporting our findings. Moreover, hypertension has been found to increase the risk of CMBs [13]. The hypertension-related pathogenesis of CMBs involves the upregulation of matrix metalloproteinases, which degrade collagen, elastin, and other components of the basal lamina and extracellular matrix, thus destroying the structural integrity of the cerebral vessels [13]. This provides evidence and explanation for the association between hypertension and CSVD. Moreover, the incidence of diabetes mellitus and the HbA1c level in our patients with CSVD was higher than in the HC group. Type 2 diabetes mellitus was found to be associated with higher risk of lacunar infarcts [14]. Georgakis et al. found that genetic predisposition to type 2 diabetes and higher HbA1c levels were associated with a higher risk of small vessel stroke, and similar associations were noted for a white matter disease marker [15]. These findings support the results of the present study. The mechanisms for this association may be related to an upregulation of inflammation. Diabetes causes the increase of vascular wall thickness, mainly hypertrophic remodeling, thus leading to the limitation of vessel reactivity and affecting cerebrovascular function [16].

Our results demonstrated that the OA diameter in patients with total CSVD scores of four was significantly lower than that in HCs, and a higher score was correlated with a smaller diameter. Owing to the small sample size, no difference in diameter was found among the CSVD subgroups. These results may be related to pathological changes in arterioles in patients with CSVD. The known causes and risk factors of CSVD include hypertension, branch atheromatous disease, cerebral amyloid angiopathy, and several genetic diseases [2]. Arterial pathologies caused by small subcortical infarcts include hyaline and hyperplastic arteriolosclerosis, which might lead to an increase in the media-to-lumen ratio. In addition, the wall of the affected cerebral small vessels thickens, and the lumen narrows because of the aggregation and deposition of abnormal proteins and collagen. We speculated that the same mechanisms were responsible for the small diameter of the OA in the patients with CSVD. Yatsuya et al. found that the narrow retinal arteriolar caliber was associated significantly with incident lacunar stroke [17]. However, the correlation between retinal arteriolar caliber and CSVD is considered controversial [17–19]. Retinal arteries are usually susceptible to hypertension, diabetes, and other factors [20, 21]. By contrast, OA can reflect changes in ocular blood flow in patients with CSVD early, directly, and accurately. Hiroki et al. reported that the end-diastolic and mean velocity of the central retinal artery were significantly lower in patients with CSVD than that in healthy controls, and the central retinal artery flow velocity was related to the severity of CSVD [22]. Similarly, our findings showed that OA blood velocity was lower in patients with CSVD than that in the HC group, and the blood flow slowed as the total CSVD score increased. For clinicians, early detection of ocular hemodynamic abnormalities can contribute to earlier detection of ocular diseases. Studies have shown that the blood flow velocity of the OA in eyes with ocular ischemia syndrome was decreased [23–25]. Ma et al. revealed that the peak systolic velocity of OA was the main indictors for the severity of ocular ischemia syndrome [23]. In this study, the blood flow velocity of the OA in the group with total CSVD score of 3 or 4 was significantly lower than that in the HC group and the group with total CSVD score of 1. We presumed that patients with total CSVD score of 3 or 4 might be at greater risk of ocular ischemia than patients with mild CSVD, because of poor ocular perfusion.

Several studies have shown that severe WMH predicts a subsequent lower cerebral blood flow [26, 27]. However, to our knowledge, there have been no reports to date on changes in ocular blood flow in patients with CSVD. Our study demonstrated that the mass flow ratio of the OA to the ipsilateral ICA decreased as the total CSVD score increased, which was a significant finding. This inspired us to pay attention to the total CSVD score in patients in clinical practice to prevent the occurrence of ocular ischemia. Unfortunately, no difference in the mass flow ratio of the OA to the ICA was elicited among the CSVD subgroups owing to the lack of sample size in each subgroup. We will use a larger sample size to further explore the association between CSVD and OA blood flow in future research.

Tarkkonen et al. reported that central retinal arteriolar equivalent was lower in patients with more than 10 CMBs than in those without [28]. The current study found that the presence of CMBs and enlarged perivascular spaces was independently related with the decrease of OA diameter, thus suggesting that the OA diameter may reflect the presence of MRI signs of CSVD.

Ischemic heart disease was independently associated with slow OA blood flow. The slow OA blood velocity is thought to be correlated with systemic atherosclerosis, including coronary atherosclerosis, which is the basic pathogenesis of ischemic heart disease [9], thus explaining the correlation between CSVD and ischemic heart disease. In addition, Novak et al. found that blood flow velocities in the middle cerebral arteries were negatively associated with the WMH volume in patients with diabetes [29]. Cerebral blood flow velocity has been found to be a strong risk factor for WMHs [30]. We found that severe WMHs was independently related to slow OA blood flow, thus suggesting that the OA blood flow velocity may become a tool for the assessment of CSVD in future studies.

This study had several limitations. First, some selection bias was introduced possibly owing to the poorer brain health of patients who had undergone brain MRI than those who had not. Second, the majority of both patients with CSVD and controls were male. Third, the slice thickness of the CTA scan influenced the accuracy of OA model reconstruction. Fourth, the sample size was small, especially in the multivariate regression analysis. Fifth, the interocular correlation was not adjusted. In addition, we set the same boundary conditions for all participants in the CFD simulation because most patients did not undergo transcranial Doppler ultrasonography, and the ICA blood flow velocity was unknown.

In conclusion, this study analyzed the OA morphological and hemodynamic changes in CSVD with the total CSVD score. Our findings indicated that the OA diameter and blood velocity in patients with CSVD were significantly lower than those in the HC group. The increase of the total CSVD score was independently associated with the decrease of the OA blood velocity. The presence of CMBs and enlarged perivascular spaces was correlated with the OA diameter, while lacunar infarcts and WMHs were correlated with the OA blood velocity. More studies are needed in the future to evaluate CSVD by observing the morphologies and hemodynamics of the OA.

## Data Availability

The datasets used and/or analyzed during the current study are available from the corresponding author on reasonable request.

## Abbreviations

CSVD: Cerebral small vessel disease
MRI: Magnetic resonance imaging
WMH: White matter hyperintensity
CMB: Cerebral microbleed
OA: Ophthalmic artery
DSA: Digital subtraction angiography
CDI: Color Doppler imaging
CTA: Computed tomographic angiography
CFD: Computational fluid dynamics
FLAIR: Fluid-attenuated inversion recovery
SWI: Susceptibility-weighted imaging
DICOM: Digital Imaging and Communications in Medicine
ICA: Internal carotid artery
HC: Healthy control
SBP: Systolic blood pressure
HbA1c: Hemoglobin A1c
